# Facilitating the coping and development of college students with mental disorders: a positive clinical psychology approach

**DOI:** 10.3389/fpsyg.2024.1373668

**Published:** 2024-06-10

**Authors:** Bo Hu, Yuxin Huang, Xinlei Yao, Chaoyang Chen

**Affiliations:** Department of Psychology, Ningbo University, Ningbo, Zhejiang, China

**Keywords:** college students with mental disorders, positive clinical psychology, resilience, optimal development, intervention strategies

## Abstract

In serving college students with mental disorders, on-campus mental health professionals have been lacking integrative theoretical frameworks to guide their missions of prevention, remedy, and development facilitation. In the current paper, we propose the positive clinical psychology as a theoretically and practically valuable framework for these missions by narratively reviewing the preventive, remedial, and developmental mechanisms derived from the theory and summarizing the most recent empirical evidence that supports each mechanism. We further discuss why and how these mechanisms and findings can be applied to on-campus mental health services to facilitate the resilience and optimal development of college students with mental disorders. Particularly, the use of resilience-focused and strength-based intervention strategies are promoted for services.

## 1 Introduction

College students are commonly experiencing the beginning of their early adulthoods, being challenged by such developmental tasks as the completion of identity development, autonomy development, and the pursuit of post-secondary education. It was estimated that over three-quarters of college students in the U.S. struggled with mental distresses with anxieties and depressive emotions being the most common symptoms (American College Health Association, 2023). Globally, the 12-month prevalence of suicidal ideation among college freshmen is 17.2% (Mortier et al., 2018). Mental illnesses have been the primary contributing factors for college students’ discontinued education (Eisenberg et al., 2009; Auerbach et al., 2016), causing immense obstacles and complex crises for students’ development.

Traditionally, the goals of mental health services on post-secondary campuses are centered on prevention, remedy, and development facilitation (Gelso and Williams, 2022). Although mental health professionals perform these missions following several different models (e.g., the medical model, the psychology model) with each focusing on one or two missions (Jensen, 2006), integrative frameworks are lacked to address the preventive, remedial, and developmental needs of college students with mental disorders (CSMD). In the past two decades, the positive clinical psychology (PCP) has exerted its clout on both research and clinical practices (Wood and Johnson, 2016). However, its rationales and usefulness in serving CSMD in the setting of post-secondary education have yet to be deliberated and reasoned. The primary goal of the current paper is to narratively review the preventive, remedial, and developmental mechanisms derived from the PCP framework and related evidence, and thereby to propose PCP as a valuable integrative framework for on-campus mental health services for CSMD. Below, we first introduce the basic tenets of PCP. We then reframe the PCP theory to explain each focused mechanism, for which we review corresponding empirical evidence. We conclude with a discussion on the applications of the mechanisms in facilitating the adaptation, coping, and development of CSMD.

## 2 The theory of positive clinical psychology

Positive clinical psychology (PCP) is conceived of to shift the focuses of existing clinical psychology into a balanced fashion, where both maladaptive and adaptive functioning are valued and learned in research and practices. In response to concerns over a separatist trend between an illness-oriented clinical psychology and a virtue-oriented positive psychology, PCP views the natures, pathologies, and remedies of mental disorders from an integrative standpoint.

Positive clinical psychology inherits some basic philosophies from its two major sources. First, in line with its positive psychology root, it views human conditions from an optimistic perspective, assuming individuals’ inherent capabilities for growth, potential fulfilment, and the development of positive adaptation. Second, PCP concentrates on the adaptiveness of a functioning, hinting its evolutionary view on human natures. That is, it simply sees humans as organisms to adapt the environment and is neutral on the nature of a functioning. Correspondingly, it opposes the illness ideology and the social construction process underlying the conception and nomenclature of mental disorders. In this lens, although harmful in general, even a maladaptive behavior can be helpful in a particular context. Lastly, PCP takes a constructivist approach to the way everyone interprets their experiences by valuing ones’ creativity in finding their own meaning and purposes out of objective conditions.

Positive clinical psychology views mental disorders as being dimensional. That is, the diagnostic criteria are simply the dimensions used to locate one’s positions on the continua of a set of characteristics with one end indicating adaptiveness and the other maladaptiveness. As such, any features that are clinically meaningful exist on continua ranging from low to high. Thus, to completely profile a mental disorder (e.g., the major depressive disorder), one’s conditions related to both “negative” features (e.g., a high sense of self-worthlessness) and “positive” ones (e.g., a low level of self-efficacy) need to be considered.

Relatedly, PCP reconceptualizes the pathologies of mental disorders by including the absence of adaptive functioning as a complement to the tradition of heavily attending to clients’ deficits in psychopathology. The idea is that not only what is going wrong (e.g., depressive or anxious emotions) but what is impeding the right (e.g., a low sense of optimism or self-efficacy) serves the causes of mental disorders, and each part works on ones’ adaptation via its distinctive route (Maddux, 2008; Lomas and VanderWeele, 2023; Zhao and Tay, 2023). Following its adaptation view, PCP challenges a traditional positive–negative dichotomy imposed to any clinical conditions. Instead, it states that the nature of a condition is determined by the context a client lives in and other conditions the client holds, and that adaptive behaviors are the results of individual–environment interactions. For the same reason, PCP advocates the use of global assessments in clinical diagnoses and evaluations.

The PCP approach to psychotherapy includes a strength-building process, through which not only can one reduce symptoms, but pursue a cure. PCP assumes a mutually inhibitory dynamic between symptom occurrence and strength building with a raise of one process leading to a fall of the other. Per PCP, building strengths and positive resources deliberately is therapeutic in that it helps lift one’s overall level of functioning despite of the maladaptation in particular domains. One’s functioning improvement enables them to meet challenges in life, activates their agency, and keeps them motivated in therapy. Also, strengthened rational and positive thoughts and (emotional and social) experiences per se are the therapeutic keys for the reduction of a variety of psychological symptoms.

## 3 The preventive, remedial, and developmental mechanisms under PCP

### 3.1 The preventive mechanism

The focus of prevention is on how to minimize the risk of developing a mental illness. PCP meets the preventive goal via increasing one’s resilience, defined as one’s capability to exert personal virtues and assets to stay adaptive under the negative effects of adversities and stresses. These virtues profile one’s characteristics of thoughts, beliefs, perception, and attribution styles, and conceptually are continua indicating two opposite directions toward adaptation and maladaptation. Thus, one’s resilience can also be seen as a continuum, contributing to the adaptative outcome, or interfering with the risk-illness chain. In facing adversities, the resilient tend to exert personal assets to actively cope with stresses, which lowers the risk; while the non-resilient take stresses passively, which exacerbates the risk (Gooding et al., 2017).

Resilience alters the risk-illness chain via two mechanisms: the compensatory process and the buffering process. The former is built upon the negative association between resilience and a mental illness, which, serving as a protective factor, offsets the effect of risk factors on the illness (Masten, 2001; Johnson et al., 2011). The latter hypothesizes the moderator role of resilience in the risk-illness link, assuming that resilience would only function when a risk is present and stay dormant otherwise. Moreover, resilience is expected to play a bigger role as the level of risk lifts.

Both processes have been evidenced. For instance, in a non-randomized clinical trial, Akeman et al. (2020) found that college students’ improvement in resilience skills (e.g., focusing on growth mindset) protected them from depressive symptoms. Similarly, early findings from re-analyzing data from Wisconsin Longitudinal Study (2006), a cohort study targeting 51- to-56-year-old adults, supported that resiliency factors (e.g., tenacity and flexibility, Kelly et al., 2013; self-acceptance, autonomy, purpose in life, and positive relationships with others, Wood and Joseph, 2010) lowered the risk of suffering depressive symptoms 10-year later, partly validating the compensatory assumption. Likewise, in a more recent cohort study (Shi et al., 2022), resilience was reported to be associated with decreased depressive and anxiety symptoms among Chinese adolescents during the COVID-19 lockdown.

Also, various resiliency factors (e.g., gratitude, flexibility, and optimism) have been shown to alter the risk-illness link in longitudinal studies. For example, Krause (2009) found that for adults over 65 years of age the association between chronic financial strain and the increase of depressive symptoms over time was conditional on the level of one’s feelings of gratitude. A greater stress-buffering effect was observed in facing more serious financial difficulties. Kleiman et al. (2013) found that a higher level of gratitude could more significantly weaken the contribution of undergraduates’ sense of hopelessness and depressive symptoms to suicidal ideations. As for other factors, Hajek and König (2021) reported that adults’ flexible goal adjustment moderates their self-rated health and negative affect. Yu et al. (2021) discovered that the association between depression profiles and suicidality among U.S. adolescents was attenuated by their levels of optimism.

### 3.2 The remedial mechanism

In PCP, the process of remedy means to pursue a cure, rather than symptom reduction per se. Particularly, PCP therapists believe that one’s mental health problems could be fixed by developing and using their strengths and by building their positive resources. Strengths has been defined as one’s built-in capacities for ways of thinking, feeling, and behaving, which allow them to achieve optimal functioning while pursuing valued outcomes (Quinlan et al., 2012). The mechanisms underlying PCP’s strength-building and positive-facilitating approach include a two-layer process. At a macrolevel, strength building helps generate positive coping experiences and personal resources, which in turn lifts one’s overall functioning and self-efficacy in coping. At a microlevel, the remedy develops via an “undoing and broaden” process (Fredrickson, 2001), which assumes that positive emotions can work on one’s symptoms by broadening the scope of one’s thoughts and behaviors narrowed by negative emotions.

At the microlevel, it has been proved that interventions focusing on facilitating strength identification and strength use are effective in enhancing clients’ agency for changes, self-efficacy, and the acquisition of personal resources (Meyers et al., 2015; Bakker and van Woerkom, 2018). Also, it has been consistently shown that strength-focused interventions can successfully boost one’s overall well-being, protect individuals from emotion problems, and facilitate personal and group gains in positive experiences and outcomes (for reviews, see Ghielen et al., 2017; Carr et al., 2021). Further, findings from studies focused on psychological capitals (e.g., Ortega-Maldonado and Salanova, 2017; Selvaraj and Bhat, 2018) suggested that the positive effect of strength interventions on mental health gains were mediated by one’s self-efficacy, hope, and optimism.

The broaden hypothesis originates from the broaden-and-build theory (Fredrickson, 1998, 2013), which hypothesizes that both negative and positive emotions are adaptively valuable with the former invokes specific cognitive and behavioral tendencies for those instant survival needs and the latter, on the contrary, widens the scope of these tendencies for long-run needs. Data coming from behavioral trials (e.g., Fredrickson and Branigan, 2005; Rowe et al., 2007), eye-tracking (e.g., Wadlinger and Isaacowitz, 2006; Lacey et al., 2021), and brain imaging (e.g., Schmitz et al., 2009) have consistently supported the hypothesis. Furthermore, it has been shown that the broadened array of cognition and action help build enduring personal resources and reduce depressive symptoms (Kok et al., 2013) and that the dynamic influencing process between positive emotions and resources proceeds in a fashion of upward spirals (Moskowitz et al., 2017; Van Cappellen et al., 2021).

### 3.3 The developmental mechanism

Positive clinical psychology assumes that development is a strength-driven process, and that the optimal development can be achieved via maximizing the benefits from one’s potentials and strengths. To maximally fulfil potentials, one needs to endeavor to spot and exert what they excel at, which has been described as a strength identification and use process (Biswas-Diener, 2010). Biswas-Diener et al. (2011) refined on the approach to focus on the dynamic growth of one’s strength competence and the importance of strength regulation. The strength development approach assumes that one’s optimal development relies on their competences in properly choosing and using strengths within varying contexts based on adaptive needs and personal values.

Research has documented that strength-based interventions that focus on strength identification and use can lead to a wide scope of positive changes in one’s psychological, social, and occupational functioning, such as meaning pursuit, well-being, work performance, and education outcomes (Ghielen et al., 2017; Quinlan et al., 2019; Dolev-Amit et al., 2021; Moore et al., 2024). Existing evidence has further shown that strength use, instead of strength identification, appears to be the active ingredient attributable for the observed positive changes in various domains (e.g., Govindjee and Linley, 2007; Wood et al., 2011; Littman-Ovadia et al., 2013; Miglianico et al., 2020). Given these recognized developmental benefits, more importantly, it has been evidenced that one’s ability to use strength can be improved through strength-based interventions (see Schutte and Malouff, 2018 for a review).

The idea behind strength development is that strength utilization is conditional on both individual and situational factors. Thus, developing the competence of strength use is essentially a process of learning how to adjust one’s characteristics, values, and interests to situational needs. Sheldon and Houser-Marko (2001) discovered that deliberate strengths use can be more productive and beneficial if it is driven by self-concordant values and goals. Besides, evidence from non-clinical populations (e.g., van Woerkom et al., 2016) has shown that extraverts and individuals with proactive personality traits at work tend to benefit more from strength use. As for situational factors, work environments that predominantly value and endorse strengths use can better help individuals engage in tasks at which they may excel and complement for each other via orchestrating everyone’s unique strengths (Moore et al., 2022).

## 4 Intervention strategies under PCP for CSMD

College students with mental disorders (CSMD) face adversities caused by dealing with the symptoms of their psychological dysfunctions, stresses from post-secondary education, and challenges in completing developmental tasks as emerging adults. From a PCP standpoint, the presence of mental illnesses complicates the challenges CSMD face in pursuing educational, developmental, and career goals, and meanwhile, provides them with opportunities to learn about and cope with their dysfunctions and to develop resilience and strengths for optimal development.

### 4.1 Facilitating CSMD’s resilience

According to Bowers (2021), optimism, ability to set and implement goals, and gratitude during suffering are the key characteristics of resilient CSMD in the face of adversities. Particularly, acceptance (i.e., openness to change) and persistence are the keys for the survival from and the success in post-secondary education despite struggles with mental health problems. Similar characteristics were also found crucial in maintaining mental health among the general population with mental illnesses (e.g., Carrillo et al., 2021; Flannigan et al., 2021; Jiang et al., 2022). Outside of optimism (or having hope), acceptance, and persistence, Edward et al. (2009) also found that the resilient lived with meaning and meaningful relationships in their lives, understood their mental illnesses, and felt a sense of universality in the context of suffering from mental illnesses.

Given these adaptive characteristics of resilient CSMD, building up such a virtue profile is a learning process. That is, to adapt to adversities, CSMD need to gain new insights into their mental health situations, lives, and themselves, to adjust their mindsets and lifestyles, and to learn new coping skills. Such a process may include formal or informal psychoeducation, effective clinical interventions, specific promotion programs for coping skills, and the advocacy of a supportive and flexible environment for learning and living. Under the PCP framework, various positive psychology intervention strategies (e.g., strength-based interventions and positive-emotion-focused interventions) are valuable additions to traditional therapeutic approaches. These strategies can be either employed separately or integrated into the therapeutic procedures of traditional approaches, such as cognitive behavioral and solution-focused approaches.

### 4.2 Facilitating CSMD’s optimal development

For CSMD, post-secondary education is an opportunity to earn academic qualifications, learn professional skills, and develop readiness for future career. However, with impaired academic and social functioning, CSMD face challenges on and off campus in their journeys to both mental health and a successful education. Given the potential benefits one can have from their post-secondary education, researchers (e.g., Schwartz, 2016; Oshri et al., 2018) viewed the educational pursuit as a turning point for resilience development if CSMD could survive and succeed in colleges. In fact, college admission success itself has showcased CSMD’s past academic achievement, strengths, and potentials for success in college. Moreover, it is a proof of one’s intelligence and capability for learning.

Per PCP, the goal of facilitating CSMD’s optimal development is to maximize the benefits of their recognized and potential strengths during post-secondary education. Identifying and using one’s strengths in the context of mental illness need additional conceptual and practical considerations. On one hand, one’s strengths and capabilities to use strength can be utilized as strategies in coping with their mental health problems. On the other hand, efforts to facilitate the maximum development of one’s strengths could help CSMD find meaning and purposes of their lives, and further lift their psychological well-being. The ideal of optimal development is that CSMD can grow and even thrive along with their symptoms.

### 4.3 PCP interventions for CSMD on college campuses

To facilitate CSMD’s mental health and success in post-secondary education, mental health professionals on college campuses need to systematically provide psychoeducation and clinical interventions and advocate a CSMD-friendly campus environment. Specifically, PCP-based interventions may be designed to focus on building CSMD’s future-oriented and gratitude mindset, on acquiring knowledge for their mental health conditions, and on learning coping skills to facilitate resilience, positive lifestyle, and strength use and development.

#### 4.3.1 Future-oriented interventions

The goal of future-oriented interventions is to help CSMD build optimistic mindsets via psychoeducation, cognitive restructuring, and the improvement of overall well-being. Optimism is essentially a cognitive-emotional construct, describing one’s tendency to think and feel positively about future. Optimistic individuals see future positively despite the adversities, and therefore are more likely engage in coping activities, such as proactively learning about their illnesses, seeking for professional helps, setting realistic therapeutic goals, and exploring pathways toward their attainment (Brissette et al., 2002; Scheier et al., 2021). More importantly, optimism is assumed to be malleable and amenable (Malouff and Schutte, 2017). For CSMD, the enhanced optimism may be part of their restructured cognition resulted from the psychotherapy they received. It may also come from trainings designed to educate CSMD to think positively.

#### 4.3.2 Strength-based interventions

Per PCP, one’s positive experiences, agency, self-efficacy, psychological well-being, and overall level of coping can be improved through maximumly developing and using their strengths. For CSMD, the development and use of strengths can also facilitate their success in post-secondary education. The context of post-secondary education is particularly valuable for strength-based intervention strategies because the strength identification, use, and development are generally valued and encouraged in educational settings. Effective strength-based interventions may need to focus on improving CSMD’s awareness, knowledge, and skills in developing and using strengths (Lechtenberger et al., 2021; Flückiger et al., 2023), as well as to advocate a strength-based interpersonal environment and campus culture. Overall, the goal of these efforts is to increase the chance for CSMD to survive and thrive in both post-secondary education and careers after college.

#### 4.3.3 Positive affect promotion and gratitude training

As a coping skill, intentionally increasing CSMD’s positive affect can effectively reduce mental illness symptoms and boost positive experiences and resources (e.g., Fredrickson et al., 2008; Kok et al., 2013). Among positive affect promotion strategies, gratitude-focused interventions (e.g., gratitude training) have been found promising in increasing CSMD’s gratitude and resilience (Bowers, 2021; Huston et al., 2024). Gratitude training may include psychoeducation for a gratitude mindset and learning how to perceive and express gratitude. Ideally, an improved ability to feel and express gratitude may help CSMD generate more positive affect, positive interpersonal interactions, social supports, and even more adaptive understanding of mental illness and the meaning of life.

## 5 Conclusion

Positive clinical psychology (PCP) provides a conceptual framework and pathways for CSMD to build resilience, reduce symptoms, and fulfil optimal development. Its resilience-focused and strength-based intervention strategies are suited for the needs of CSMD as the post-secondary education and PCP interventions share common focuses on CSMD’s growth and development. For the same reason, in an ideal educational environment, the post-secondary education itself can be seen as a great opportunity for CSMD to learn coping skills and develop adaptive functioning. In practice, mental health professionals on campuses are suggested making further efforts in recognizing and utilizing their clients’ strengths and intellectual advantages, as well as the potential resources and supports from an educational environment. Future studies are needed to identify the obstacles to practicing the PCP approach in on-campus mental health services. Efforts are also needed to integrate PCP-based strategies into traditional intervention approaches, and further to evaluate the efficacy of each endeavor.

## Author contributions

BH: Writing−original draft. YH: Writing−review and editing, Project administration. XY: Writing−review and editing, Project administration. CC: Writing−review and editing, Conceptualization.
